# Comparative analysis of genome code complexity and manufacturability with engineering benchmarks

**DOI:** 10.1038/s41598-022-06723-5

**Published:** 2022-02-18

**Authors:** Joseph Riolo, Andrew J. Steckl

**Affiliations:** Nanoelectronics Laboratory, Department of Electrical Engineering and Computer Science, University of Cincinnati, Cincinnati, OH 45221-0030 USA

**Keywords:** Engineering, Biotechnology, Industrial microbiology, Software

## Abstract

When knowledge has advanced to a state that includes a predictive understanding of the relationship between genome sequence and organism phenotype it will be possible for future engineers to design and produce synthetic organisms. However, the possibility of synthetic biology does not necessarily guarantee its feasibility, in much the same way that the possibility of a brute force attack fails to ensure the timely breaking of robust encryption. The size and range of natural genomes, from a few million base pairs for bacteria to over 100 billion base pairs for some plants, suggests it is necessary to evaluate the practical limits of designing genomes of similar complexity. This analysis characterizes the complexity of natural genomes, compares them to existing engineering benchmarks, and shows that existing large software programs are on similar scale with the genome of complex natural organisms.

## Introduction

It took a hundred years from the advent of the term “synthetic biology”^1,2^ to the modern confirmation of its essential technologies when, in 2010, researchers at the J. Craig Venter Institute (JCVI) created a synthetic *Mycoplasma mycoides* genome and transplanted it into a recipient *Mycoplasma capricolum* cell^3^. *Mycoplasma genitalium* was originally selected because, at the time, it had the fewest genes of any known organism capable of independent growth but *Mycoplasma mycoides* and *Mycoplasma capricolum* were later selected for their faster growth rate. The creation of a viable bacteria cell controlled by a synthetic genome (JCVI-syn1.0) ushered in an era where scientists and engineers intend to produce artificial life forms for their own purposes and represented a major break from genetic engineering that was initially focused on modifying existing organisms. Synthetic biology is a discipline that “uses engineering principles to design and assemble biological components”^4^. Simply stated, synthetic biology aims to use the knowledge that sequencing of the genome of species has provided (the biological code) to manufacture biological components. Synthetic biology has the potential to be the next epochal technological human advancement following microelectronics and the internet. The potential of synthetic biology has been recognized by many organizations, from being highlighted as a top technology by the World Economic Forum^5^ to being included on the list of "Big Ideas for Future Investments" of the US National Science Foundation (NSF)^6^. This high level of emphasis, including major research stimulus by NSF in 2019 to understand the “Rules of Life”^7^, has resulted in significant practical and academic advancements, such as genome rewriting for streamlined synthesis^8^.

**Figure 1:**
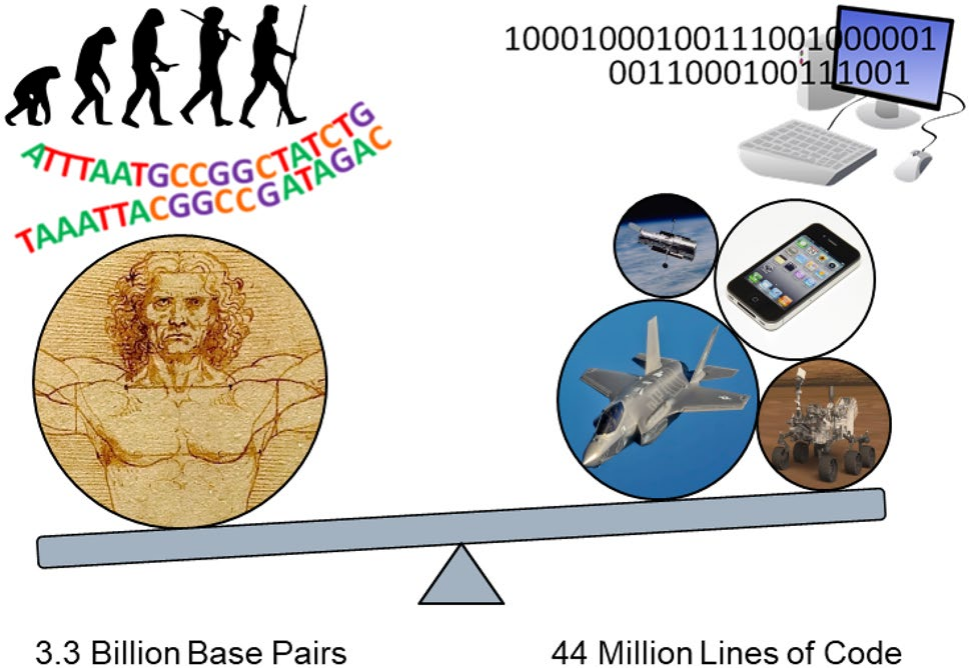
Comparison of complexity between the human genome and several major engineering systems. Image credits: Hubble and Mars Curiosity Rover: NASA/JPL-Caltech; iPhone: Yutaka Tsutano (CC BY 2.0), F-35: MSgt Donald Allen; March of Progress: M. GardeFerdinand (CC BY-SA 3.0).

Based on his experiences Venter observed that the “genome design's greatest limitation” is the lack of fundamental knowledge^9^. It is unclear at this time if a predictive understanding of organism phenotype will ever be realized but, if the necessary knowledge is gained, it seems likely that practical applications of synthetic biology would be enabled by technologies such as abstraction, standardization, and decoupling^10^. The presence of fundamental knowledge and the availability of appropriate tools does not necessarily guarantee the creation of useful synthetic organisms since it has not yet been demonstrated that complexity itself is not a limitation. Is it practical to design large scale synthetic organisms or is comprehensive genome design too complex for economic usefulness? The objective of this analysis is to begin a discussion on the feasibility of potential synthetic biology undertakings by establishing metrics against which genome complexity and manufacturability can be measured. Software (the current human coding champion) in its compiled form and semiconductor manufacturing have been selected as the yardsticks for comparison. Large scale software programs are increasingly being developed for all aspects of life from automotive entertainment to aerospace controls and financial systems to weather prediction. Indeed, with the advent of the “internet-of-things”, digital programming is destined to become the ubiquitous communication and control sinew of mankind. While the study presented here is not quantitative in the sense of providing an expected timeline for synthetic biology breakthroughs it may provide a qualitative assessment of feasibility in a manner similar to how a brute force encryption vulnerability assessment would determine if a key could be broken in a reasonable time^11^. As illustrated in Fig. 1, our analysis indicates a rough equivalence in complexity between various major engineering systems and the human genome.

## Results

### Selecting suitable benchmarks for measuring genome complexity

The sequence of nucleotides in a genome is commonly referred to as its genetic code which, for electrical and software engineers, is immediately reminiscent of computer object code. Object code is the output of a compiler that translates source code in a high-level programming language into machine useable binary instructions. The characterization of genetic and binary digital information as ‘code’ may make comparisons seem superficial^12^. However, there are meaningful similarities that make software a suitable benchmark and, at the least, more promising than alternatives^13^. For example, both types of code store data in limited serial instruction sets (quaternary or binary) and can produce arbitrarily complex outcomes when executed on deterministic systems. These similarities were not wasted on Venter who observed that “DNA is the software of the cell”^9^. Indeed, the programming and self-assembling functions of DNA have generated great interest for high density information storage^14^, biological computing^15,16^, and nanostructure formation^17,18^. Further, the unusual electronic and optical properties of DNA thin films have been utilized in a variety of device applications^19,20^.

It is important to point out at the outset two key differences between DNA coding and digital coding. First, DNA coding regions represent a small fraction of the overall polymer. However, this coding fraction varies among species, and it also changes with time as new roles for sections of the supposedly non-coding regions are being discovered. In this initial assessment of biological vs digital coding including the entire DNA sequence in the comparison has the advantage of working with “hard” numbers. Furthermore, this approach establishes the upper bound of biological complexity, with the possibility of reducing that level as DNA portions are confirmed to have no coding significance. Second, the functionality of bioorganisms can be described as bottom-up, distributed, self-replicating, and non-deterministic, whereas computer system design and functionality is top-down, concentrated, not self-replicating (yet), and deterministic. For example, the entire machinery for self-replication and functioning of an *E. coli* bacterium is contained in a volume of one femtoliter. Research is being pursued to create self-replicating machines, which could be a more complete analog to biological systems. However, at this time they are far from the point where a comparison with biological systems would be meaningful. It will be interesting to see if advances in synthetic biology will inform our design of artificial systems in the future to be more encapsulated.

**Figure 2:**
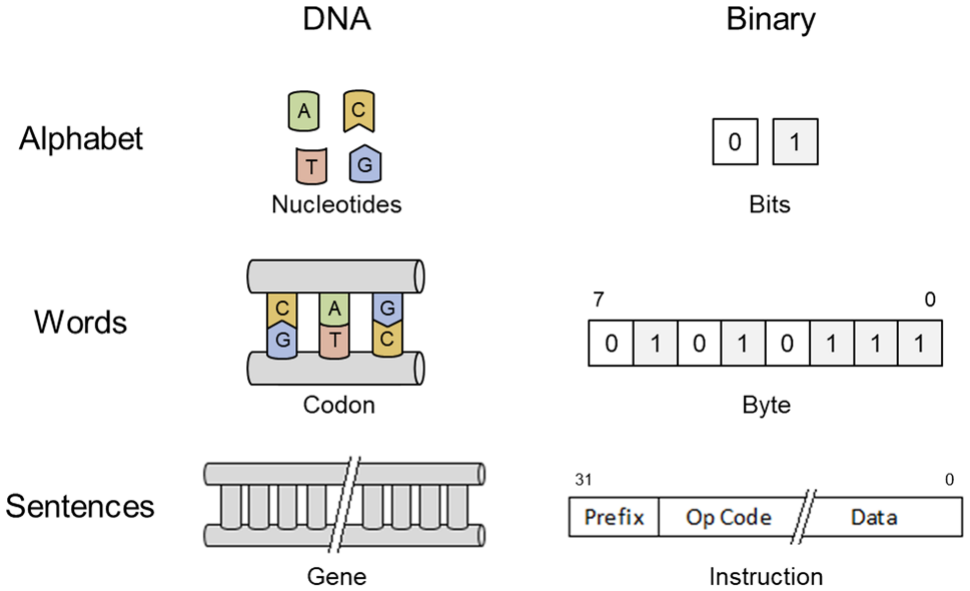
Comparison of biological and digital languages.

Figure 2 explores the parallels between genetic code and object code. Each language is based on a limited alphabet (nucleobases and bits) from which words are constructed (codons and bytes) leading to higher level organizations (genes and instruction sets). Additional similarities include context dependence, non-obvious regions of code, and resiliency to errors. Software is generally considered context independent due to the abstract nature of high-level languages and the arbitrary order that functional segments may be provided to compilers, but the resulting object code is highly context and platform dependent. Without complete understanding of the object code, making changes to individual bits, or even encapsulated functions, would present challenges like those experienced by current DNA designers attempting to manipulate base pairs or sequences. Instructions would be identified first, similar to our understanding of coding DNA, but compiled object code would also have non-obvious regions like graphical textures, audio files, or other large data sets that would be as difficult to reverse engineer as noncoding DNA. Programs even have an analog to the C-Value enigma, the observation that genome size varies largely between species and has no relationship to the organism’s presumed complexity, with recent program memory requirements exploding despite having similar functional complexity. For example, one game might even vary in installed size by nearly 200% (17–49 GB) depending on the platform due to the difference in audio file compression^21^. DNA and binary code are also susceptible to errors, which occur at an average rate of 1.28 × 10^–8^ mutations per nucleotide per generation for humans^22^ and 1 × 10^–14^ nonrecoverable read errors per bits read for hard drives^23,24^. These rates are not directly comparable, but they do necessitate appropriate error detection, correction, and redundancy to be built into both biological and digital systems.

While this equivalence between the components of DNA molecules and computer instructions may be an oversimplification, it at least provides a starting point for further thought and future more detailed comparisons. Even those that object to the metaphor for semantic reasons find the associated tools to be “remarkably useful in practice”^25^. It is interesting to point out that nature has developed the genetic code for each organism and has provided the built-in mechanisms for its replication and conversion into function. On the other hand, digital software designed for each specific application requires a separate hardware design and fabrication for conversion to a desired set of functions. However, here too we can see some similarities in that one can identify the alphabet of electronics (resistors, capacitors, transistors) leading to words (memory cells, amplifiers, etc.) and sentences (shift registers, etc.) that are joined into complex functioning systems (computers, phones, etc.). The evolution of the integrated circuit (Moore’s law) provides another useful yardstick for comparison between the development of electronics technology and the technology for genome sequencing and synthesis.

**Figure 3:**
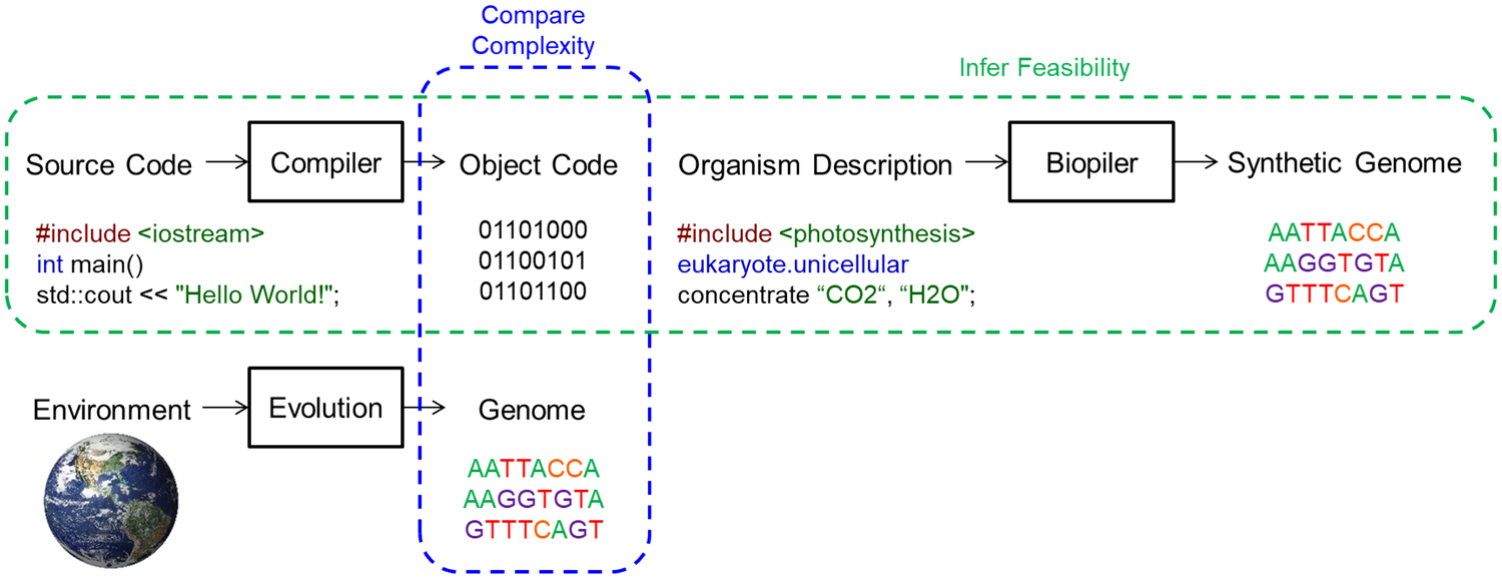
Framework for inferring feasibility of synthetic biology by comparing software object code and organism genome complexity with associated examples or hypothetical implementations. Image of Earth credit: NASA.

In addition to the similarities between the genome and the object code (binary) produced by a compiler, there are likely similarities to future processes for use in the design of synthetic organisms. Specifically, it seems likely that a high level abstract organism description language will be necessary along with a biological “compiler” to facilitate designs and translate the designers’ intent into a synthetic genome. This organization, shown schematically in Fig. 3, suggests a framework by which observable complexities in natural organisms and large scale software projects might be used to infer the feasibility of potential synthetic organism design efforts. Figure 3 also contains a simple example of high level software code being compiled into object code and explores how future tools might be used to design synthetic genomes based on desired phenotype. Synthetic biologists in training may start by coding simple algae similarly to novice software designers starting with the message “Hello world!”.

### Comparing genome complexity to benchmarks

The combinatorial complexity of a string of binary values, *C*_*binary*_, is calculated using the number of bits in the string, *N*_*bits*_ (Eq. 1). Using digital combinatorial complexity as a model, genome complexity, *C*_*genome*_, is quantified by calculating an equivalent byte value with the number of base pairs, *N*_*bp*_. Since there are four natural nucleobases, each pair of bases (cytosine [C], guanine [G], adenine [A], and thymine [T]) has a combinatorial complexity equivalent to two bits (Eq. 2). This can then be converted to bytes (and higher order units like gigabytes) to easily understand the magnitude of a genome’s complexity. Equations (1) and (2) can be combined to derive the relationship between binary and genome complexity (Eq. 3) as well as the associated ratio of bits and base pairs (Eq. 4). Additionally, one byte is composed of eight bits (Eq. 5).

1 $$C_{binary} = 2^{{N_{bits} }}$$

2 $$C_{genome} = 4^{{N_{bp} }}$$

3 $$C_{genome} \;(N_{bp} ) = C_{binary} \;(2N_{bits} )$$

4 $$N_{bp} = 2N_{bits}$$

5 $$4N_{bp} = N_{bytes}$$

This approach makes genome complexity easily quantified in terms of stored information, or equivalent bytes, based on the number of base pairs. Paradoxically, the corresponding software object code complexity is more difficult to calculate because most historical engineering projects do not have documentation available for file size or physical memory requirements. Typically, the information available is mainly on the number of Lines of Code (LOC). One study, using the C programming language, reported an average of 17 ± 3.5 bytes per line of code^26^. While this is only one study with one language that is not necessarily inclusive of all benchmarks, C is a very common programming language and will provide a qualitative comparison. A wide range of software benchmarks were selected from available sources across industries, scope, production volume, and time to yield the best opportunity for finding equivalent complexity with naturally occurring genomes.

**Figure 4:**
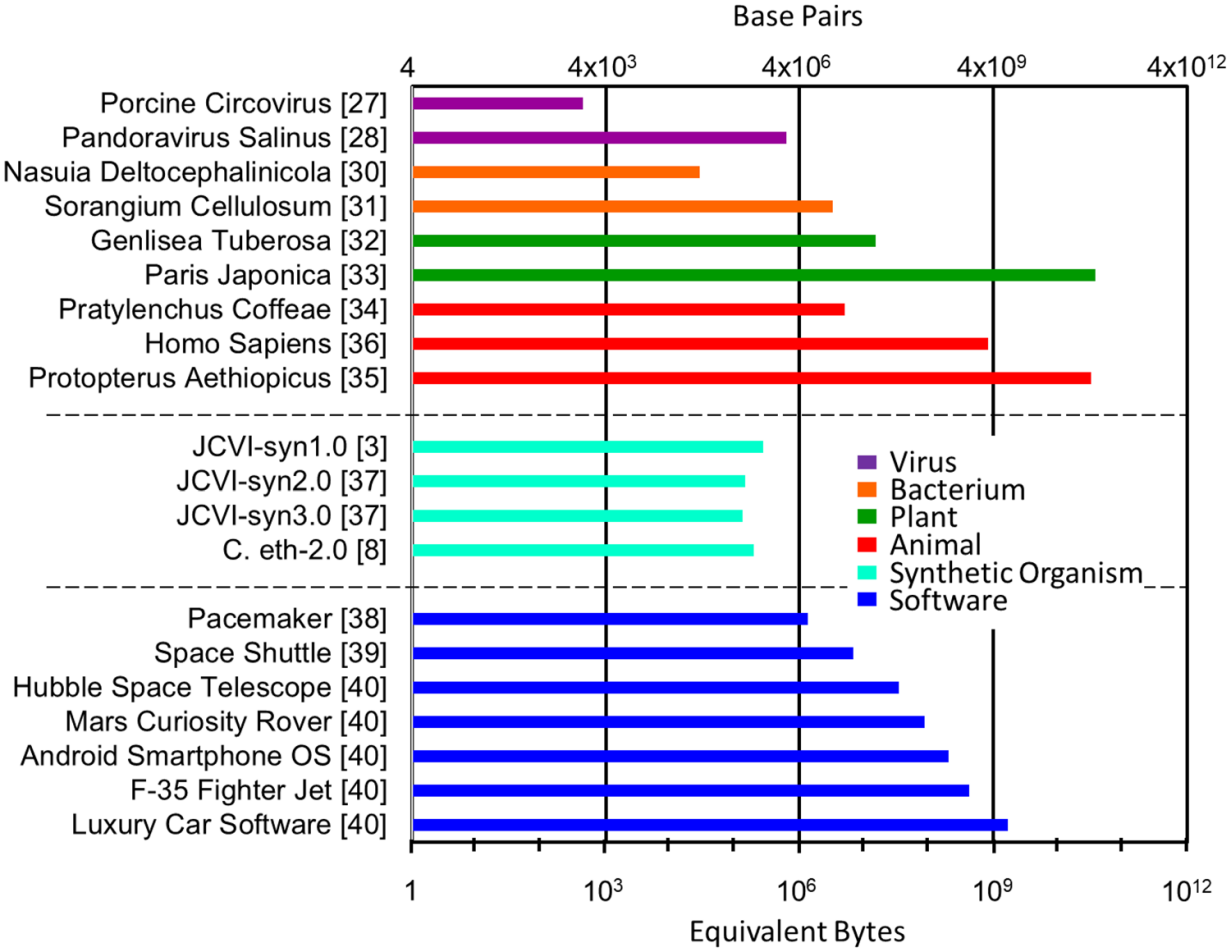
Complexity of selected natural organisms (in terms of base pairs) and software benchmarks (in terms of equivalent bytes)^3,8,27,28,30–40^.

A wide range is necessary since natural genomes vary from as few as thousands of base pairs for some viruses, to millions of base pairs for bacteria, and even as high as 100 billion base pairs for some plants. The resulting combinatorial complexity of representative examples of natural genomes and selected digital benchmarks is shown in Fig. 4. The *Porcine circovirus*^27^ has one of the smallest genomes at ~ 1800 bases (not base pairs in this case since it consists of a circular single-stranded DNA molecule in contrast to the other genome examples which are double stranded DNA molecules). The genome of the *Pandoravirus salinus*^28^ is an example of one the larger virus genomes at ~ 2.5 Mbp. By comparison, the genome of the new coronavirus responsible for the COVID 19 pandemic contains 29 kbp^29^. *Nasuia deltocephalinicola*, a bacterium that helps certain insects synthesize essential amino acids has the smallest genome of any cellular organism sequenced to date^30^, has just ~ 100 kbp, whereas *Sorangium cellulosum*, a cellulose degrading bacterium, has a circular DNA genome^31^ of ~ 13 Mbp. In the plant category, *Genlisea tuberosa* has the smallest genome reported^32^ at 61 Mbp, while *Paris japonica*, a plant native to the mountainous regions of Japan, contains possibly the largest known genome^33^ of all living species at ~ 150 Gbp. In the animal kingdom, the nematode *Pratylenchus coffeae* has one of the smaller genomes^34^ of 20 Mbp, while *Protopterus aethiopicus*, the marbled lungfish, comes in a close second to *P. japonica* for the largest genome^35^ found to date of ~ 130 Gbp. Finally, the genome of our own species^36^, *Homo sapiens*, weighs in at ~ 3.3 Gbp. While this is a very large genome, it is not the largest of either animal or plant species. As indicated above, the genome of *P. japonica* is ~ 50 × larger.

The relative complexity of several synthetic organisms is also shown for comparison. JCVI-syn1.0, the first reported cell with a chemically synthesized genome, contains approximately 1 Mbp^3^ and is a modified version of the *Mycoplasma mycoides* bacterium with synthetic ‘watermark’ sequences to differentiate from the natural genome. The synthetic *M. mycoides* cells were reported to have the phenotypic properties of natural cells and to be able self-replicate. Researchers at JCVI minimized the *Mycoplasma mycoides* genome further and tested the effects of reorganization on JCVI-syn2.0 with 580 Kbp and JCVI-syn3.0 with 530 Kbp. While all of the JCVI species were viable the simpler forms did demonstrate reduced growth rate with a doubling time of 60, 92, and 180 min for syn1.0, syn2.0 and syn3.0, respectively^37^. *Caulobacter ethensis*-2.0 (C. eth-2.0) is the product of recent research at the Institute of Molecular Systems Biology in Zurich, Switzerland that demonstrated synthesis optimized sequence rewriting on *Caulobacter crescentus*, a widely distributed fresh water bacteria and common cell cycle model^8^. Interestingly, even JCVI-syn1.0, the largest synthetic genome to date, is only about 20% the complexity of the pacemaker (4 × 10^4^ LOC)^38^ which is the simplest benchmark at just over 1 MB equivalent.

In addition to the pacemaker, several engineering projects with significant software content (for their time) were selected to act as benchmarks for the complexity of natural and synthetic organisms. The next three more complex projects in terms of lines of code being the Space Shuttle flight control (4 × 10^5^ LOC)^39^, the Hubble Telescope (2 × 10^6^ LOC)^40^, and the Mars Curiosity Rover (5 × 10^6^ LOC)^40^, corresponding to 6.8, 34, and 85 MB equivalent, respectively. These space applications range in time but their software complexity is somewhat limited based on the harsh radiation environment and high reliability requirements. The F-35 environment, which is perhaps not as harsh are space, still results in severe requirements limiting software size. However, more recent technology and emphasis on the ‘digital plane’ have resulted in a flight control software complexity of 25 million lines of code^40^ or 425 MB equivalent. Lastly, consumer electronics including cars and phones have experienced an explosion in functionality and interconnectivity that have driven their software complexity up to the point where your smart phone with its Android OS (1.2 × 10^7^ LOC)^40^ can connect to your car (1 × 10^8^ LOC)^40^ with equivalent complexities of 204 MB and 1.7 GB, respectively. This level of complexity is enabled by the mass production and ubiquitousness of these products and will continue to grow with the desire for more integration.

The similarity in complexity between software projects and natural genomes suggests that designing complex synthetic biosystems, while quite challenging and requiring major effort, will be feasible once the relationship between genotype and phenotype is well understood. Unfortunately, the ad hoc process that has been utilized for most synthetic biology research is still the standard mode for current efforts^10^. As important as the creation of the 531 kbp JCVI-syn3.0 minimal bacterial genome was in 2016^37^, the method of arriving at the genetic sequence was empirical and a key factor in the selection of the target organism (*M. mycoides*) was previous experience in synthesizing its complete genome (JCVI-syn1.0)^3^. The design process is in its infancy but the work on JCVI-syn3.0 showed that reorganizing modularized genome segments did not observably affect the resulting cells, which suggests that the development of standard parts by organizations like iGEM^41^ could find wide applicability.

This first order analysis attempts to provide a qualitative evaluation of genome complexity but does not consider all known complexities and cannot consider future breakthroughs on the path to complete understanding of the relationship between phenotype and genotype. Therefore, the complexity of natural organisms may be somewhat understated because of the absence of epigenetic factors like DNA methylation and histone modification. As advanced sequencing technologies, for example single molecule real time (SMRT), provide additional information in parallel with the primary DNA sequence^42^ these nuances should be weighed against potential efficiencies, such as the significant reduction in base pairs observed between JCVI-syn1.0 to JCVI-syn3.0. It will be interesting to see if human ingenuity can expand the accessibility of synthetic biology through simplification or if mitigating efforts will be overcome by our increasing understanding of the complexities of fundamental biological processes.

### Evaluating manufacturability of synthetic biology products

Since the 1st order comparison study indicates that designing synthetic organisms that approach the complexity of natural examples would be of a similar order of magnitude to existing engineering projects the remaining considerations would be engineering issues, such as manufacturing methods and cost. After the non-recurring engineering costs necessary to design a synthetic organism, the next most significant will be gene sequencing and synthesis. Gene sequencing is the process of determining the order of nucleotides in DNA and gene synthesis is the process of creating short oligonucleotide fragments and assembling them into a complete genome. Gene synthesis also requires many intermediate error-checking and correcting steps that involve multiple sequencing operations. Costs for both processes must be considered since a prohibitively high estimate for a complex genome could negatively impact the practicality of synthetic biology.

**Figure 5:**
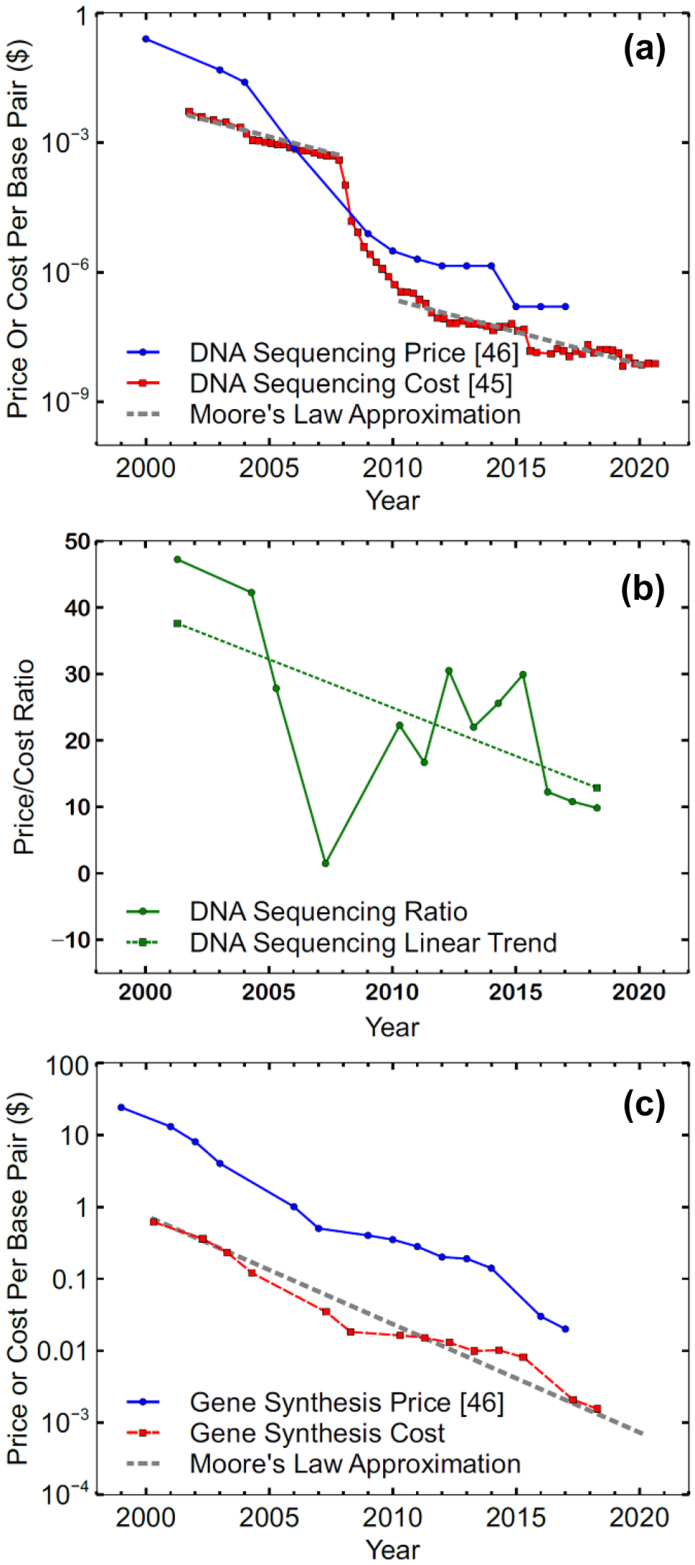
Price and cost of DNA sequencing for the 2000–2020 time period (**a**); price/cost ratio for DNA sequencing (**b**); price and cost of DNA synthesis (**c**) and comparison to an approximation of Moore’s Law^45,46^.

In addition to the functional similarities between software and genetic code, the sequencing and synthesis of genes also appear to be benefiting from significant reductions in cost as a function of time similar to the advancement of semiconductor circuit (“chip”) fabrication that is described as Moore’s Law, namely the doubling of transistors per chip every 1.5–2 years while the overall chip cost per unit area has remained more or less constant^43^. This empirical relationship is consistently reported as about to expire, but to date it has achieved a nearly 50-year validity. A corollary of Moore’s Law indicates a roughly halving of cost per transistor every 2 years^43^. As shown in Fig. 5a, DNA sequencing price and cost have closely followed this trend since 2000 except during 2008 when sequencing cost dropped precipitously as researchers worked toward the goal of a $1000 human genome to facilitate cost-effective personal diagnostics^44^.

Price and cost are often used interchangeably in everyday language, but the distinction is critical when considering production strategies. Cost is the expense incurred by a company to produce and sell a product or service and price is the amount a customer is willing to pay for that product or service. Figure 5b shows the sequencing price/cost ratio for the two decades from 2000 to 2020. Also shown in Fig. 5b is an extracted simplified time-dependent linear relationship that was used in conjunction with synthesis price data to estimate a synthesis cost in order to obtain a complete picture of the expenses related to sequencing and synthesis. Synthesis price and an estimate of cost are shown in Fig. 5c. The synthesis cost is important for consideration, but its classification as an estimate is important since Fig. 5a,b show that there may be a discrepancy between the data sets causing sequencing price to fall below sequencing cost in the 2006–2009 timeframe or the data may faithfully show temporary market conditions driven by liquidation, oversupply, or other factors. While price and cost can vary depending on method and number of nucleobases it will be important to consider both depending on application just like current microchip designers much choose to fabricate their devices or purchase wafers from an independent manufacturer. It will also be interesting to see if the DNA sequencing price and cost continue to converge, with the ratio of the two falling from 47 in 2001 to ~ 10 in 2018, as technology advances and commoditizes the industry.

## Discussion

**Figure 6:**
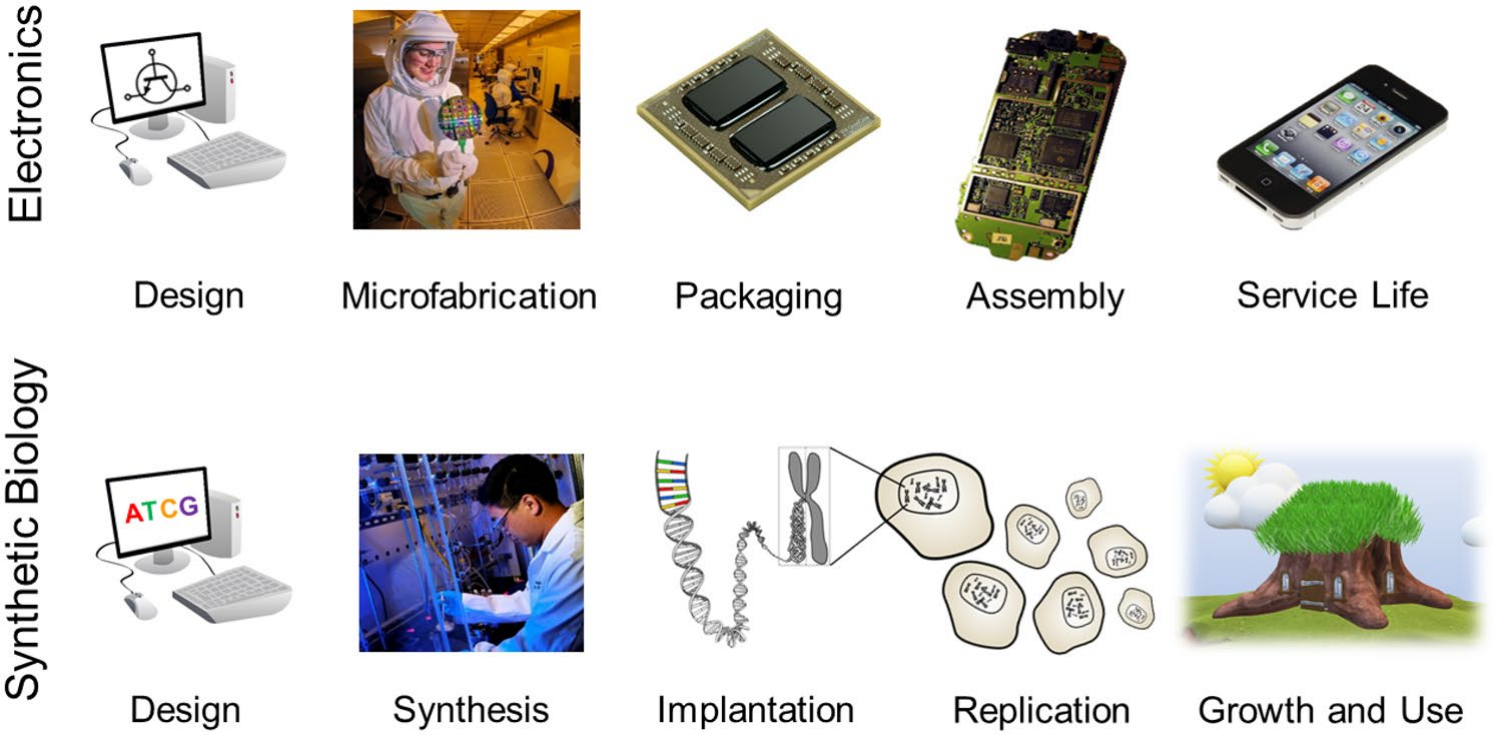
Comparison of life stages of consumer electronics and synthetic biology products. Image credits: Microfabrication: Sandia Labs (CC BY-NC-ND 2.0); Packaging: viagallery.com (CC BY 2.0); Assembly: Uwe Hermann (CC BY-SA 2.0); Service Life: Yutaka Tsutano (CC BY 2.0); Synthesis: Oak Ridge Lab News (CC BY 2.0).

With the costs of gene sequencing and synthesis dropping precipitously the main manufacturing challenge is the manual gene assembly, isolation, and transplantation processes used for current projects. Automation of these processes may be complex, but overall, the manufacturing needs for synthetic biology appear to be similar to those used for the fabrication of consumer electronics. The similarities to micro fabrication suggest processes where a large non-recurring engineering investment up front leads to high throughput, inexpensive manufacturing. Synthetic biology also has the benefit of being self-replicating, which could provide additional benefit over microfabrication if it can be adapted to contemporary lean manufacturing principles like takt, or cycle, time^47,48^. These parallels and a potential process overview of a synthetic biology factory of the future are shown in Fig. 6. Alternatively, if long gestation and/or adolescent periods seen in complex natural organisms cannot be overcome and production steps remain long like additive manufacturing, then there may be opportunities for new manufacturing philosophies that better utilize batch production^49^.

The comparison of complexity between natural species and major software endeavors makes it clear that designing synthetic organisms will be feasible when the fundamental knowledge becomes available. If synthesis costs continue to shrink at a rate similar to transistor cost as described by Moore’s law, it is also likely that the cost to synthesize genomes of equivalent complexity to even the largest genomes observed in natural organisms will be within the reach of government, commercial, and perhaps research organizations by the time this genome/phenotype linkage is fully mapped out. Advancements in the genome design may even spur additional genome synthesis cost reductions similar to those seen in sequencing during the 2008–2010 period. Extrapolation of the Moore’s Law gene synthesis estimation shown in Fig. 5c would predict a cost of $0.0003 per base pair in the 2021–2022 time period. This would mean that synthesizing an artificial human genome (~ 3.3 Bbp) would cost approximately one million dollars and simpler applications like a custom bacterium (~ 13 Mbp) could be synthesized for as little as $4000. This combination of surmountable complexity and moderate cost justifies the academic enthusiasm for synthetic biology and will continue to inspire interest in the rules of life.
